# Nutrient Condition in the Microenvironment Determines Essential Metabolisms of CD8^+^ T Cells for Enhanced IFNγ Production by Metformin

**DOI:** 10.3389/fimmu.2022.864225

**Published:** 2022-06-29

**Authors:** Ruoyu Chao, Mikako Nishida, Nahoko Yamashita, Miho Tokumasu, Weiyang Zhao, Ikuru Kudo, Heiichiro Udono

**Affiliations:** Department of Immunology, Okayama University Graduate School of Medicine, Dentistry, and Pharmaceutical Sciences, Okayama, Japan

**Keywords:** CD8+ T lymphocytes, glycolysis, FAO, glutaminolysis, IFNg, autophagy +T, metformin

## Abstract

Metformin (Met), a first-line drug for type 2 diabetes, lowers blood glucose levels by suppressing gluconeogenesis in the liver, presumably through the liver kinase B1-dependent activation of AMP-activated protein kinase (AMPK) after inhibiting respiratory chain complex I. Met is also implicated as a drug to be repurposed for cancers; its mechanism is believed identical to that of gluconeogenesis inhibition. However, AMPK activation requires high Met concentrations at more than 1 mM, which are unachievable *in vivo*. The immune-mediated antitumor response might be the case in a low dose Met. Thus, we proposed activating or expanding tumor-infiltrating CD8^+^ T cells (CD8TILs) in a mouse model by orally administering Met in free drinking water. Here we showed that Met, at around 10 μM and a physiologically relevant concentration, enhanced production of IFNγ,TNFα and expression of CD25 of CD8^+^ T cells upon TCR stimulation. Under a glucose-rich condition, glycolysis was exclusively involved in enhancing IFNγ production. Under a low-glucose condition, fatty acid oxidation or autophagy-dependent glutaminolysis, or both, was also involved. Moreover, phosphoenolpyruvate carboxykinase 1 (PCK1), converting oxaloacetate to phosphoenolpyruvate, became essential. Importantly, the enhanced IFNγ production was blocked by a mitochondrial ROS scavenger and not by an inhibitor of AMPK. In addition, IFNγ production by CD8TILs relied on pyruvate translocation to the mitochondria and PCK1. Our results revealed a direct effect of Met on IFNγ production of CD8^+^ T cells that was dependent on differential metabolic pathways and determined by nutrient conditions in the microenvironment.

## Introduction

Cancer incidence and mortality are significantly improved in patients with diabetes who take metformin (Met) for a long period compared with those taking other anti-diabetes drugs (1–3). As a result, the antineoplastic effect of Met has received increasing attention. However, the precise mechanism of the Met-dependent antineoplastic effect is highly controversial, according to current research. As respiratory chain complex I is a target of Met (4, 5), the inhibition of oxidative phosphorylation (OxPhos) followed by the activation of liver kinase B1 (LKB1)/AMP-activated protein kinase (AMPK) axis may downregulate mTORC1 in tumor cells (6–8). However, this strategy requires treatment with Met at the mM range, which is practically impossible to achieve in the clinical setting. For example, according to a previous study, Met concentrations in plasma and tumor are both in the range of 3.2 to12.4 μM when the mice receive 1.25 mg/mL Met in free drinking water (9); the dosage of Met is close to that for the patients with diabetes receiving Met. Usually, these concentrations of Met do not inhibit tumor growth *in vitro*.

Previously, we reported that Met administration *via* free drinking water rendered the mice to reject once established, highly immunogenic solid RL1 tumor (10). This phenomenon was mediated by CD8 T^+^ cells as the injection of an anti-CD8 monoclonal antibody (mAb) abolished Met’s antitumor effect. A similar effect was observed in other tumor models, such as Renca (renal cell carcinoma), and a smaller effect in four other tumor models (10). Moreover, we recently demonstrated that Met-induced mitochondrial ROS (mtROS) stimulated both Glut-1expression on cell surface of tumor-infiltrating CD8^+^ T cells (CD8TILs) to produce IFNγ and activation of Nrf2/mTORC1/p62 axis for CD8TILs to proliferate in tumor (11). In another study, Met was shown to enhance the effect of the cancer immunotherapy of PD-1 blockade in the MC38 (colon carcinoma) model; the effect was attributed to the improvement in tumor hypoxia following the OxPhos inhibition in tumor cells by Met (12). However, the authors did not propose a direct effect of Met on CD8TILs. In addition to the immune-modulatory effect of Met, there is evidence for the non-immune-mediated antineoplastic effect by biguanides, including phenformin, in immune-deficient mice (13, 14). Indeed, we also observed Met’s antitumor effect against osteosarcoma in SCID mice; however, the effect was canceled by the injection of anti-CD11b mAb (15), suggesting the involvement of M1-like macrophages or NK cells but not T lymphocytes in certain tumor cells. Meanwhile, other studies suggest that Met’s antineoplastic effect is attributed to the downmodulation of PD-L1 on tumor cells *via* its degradation in the endoplasmic reticulum (ER) (16–18). Lastly, a recent study suggests that Met enhances an antitumor vaccine’s effect by reducing the PD-L1 levels on tumor cells (19).

The synthesis of the transporters for Met, such as organic cation transporter 1(OCT-1), is critical for the effect of Met. We recently found that protein expressions of OCT-1 and glucose transporter-1 (Glut-1) were substantially enhanced *via* the stimulation of TCR using anti-CD3 and anti-CD28 antibodies (Abs), although these genes were much less expressed in unstimulated splenic CD8^+^ T cells (11). Therefore, it is necessary to investigate whether CD8^+^ T cell functions, such as IFNγ production, are enhanced by Met at a low concentration. The current study revealed the direct effect of Met at 10 μM on IFNγ production by CD8^+^ T cells and that the essential metabolism varied under different nutrient conditions in the microenvironment.

## Results

### Met at 1 mM or Higher Downregulates Oxygen Consumption Rate (OCR) While Upregulates ECAR in Tumor Cells

**Figure 1:**
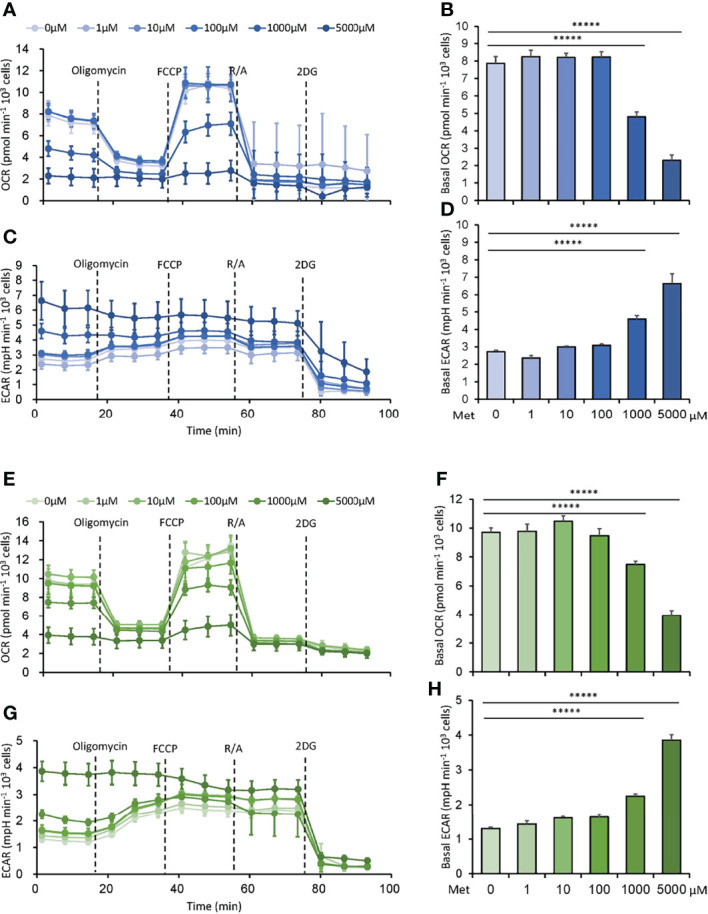
Met at 1 mM or higher downregulates oxygen consumption rate (OCR) while upregulates extracellular acidification rate (ECAR) in tumor cells. MO5 and 3LL cells were cultured *in vitro* with the indicated concentrations of Met for 48 h before the extracellular flux analysis. **(A–D)** The metabolic characteristics of the MO5 cells showing **(A)** OCR, **(B)** basal OCR, **(C)** ECAR and **(D)** basal ECAR levels. **(E–H)** The metabolic characteristics of the 3LL cells showing **(E)** OCR, **(F)** basal OCR, **(G)** ECAR and **(H)** basal ECAR levels. The graphs represented Mean ± SEM of the results of three independent experiments. Statistical analysis was performed by unpaired, two-tailed Student’s t-test (*****P ≤ 0.00001).

First, we explored the dose-response relationship between Met and the metabolism of tumor cells. Murine MO5 (B16 melanoma expressing ovalbumin) and 3LL (lung adenocarcinoma) cells were cultured with various doses of Met at 0–5 mM for 48h. Then, OCR and ECAR were simultaneously examined using a Seahorse flux Analyzer. Consistent with previous studies, OCR was inhibited by Met at 1 mM or higher in both cell lines (**Figures 1A, B, E, F**). Surprisingly, ECAR, indicating glycolysis, was elevated by Met at 1 mM or higher in a dose-dependent manner (**Figures 1C, D, G, H**), suggesting an acceleration of the Warburg effect in the progression of oxidative stress due to Met at 1 mM or higher. In any case, physiologically relevant concentration of Met below 100 μM did not affect the metabolic profile of tumor cells.

### Less Than 100 μM Met Enhances IFNγ Production by CD8^+^ T Cells Upon TCR Stimulation

**Figure 2:**
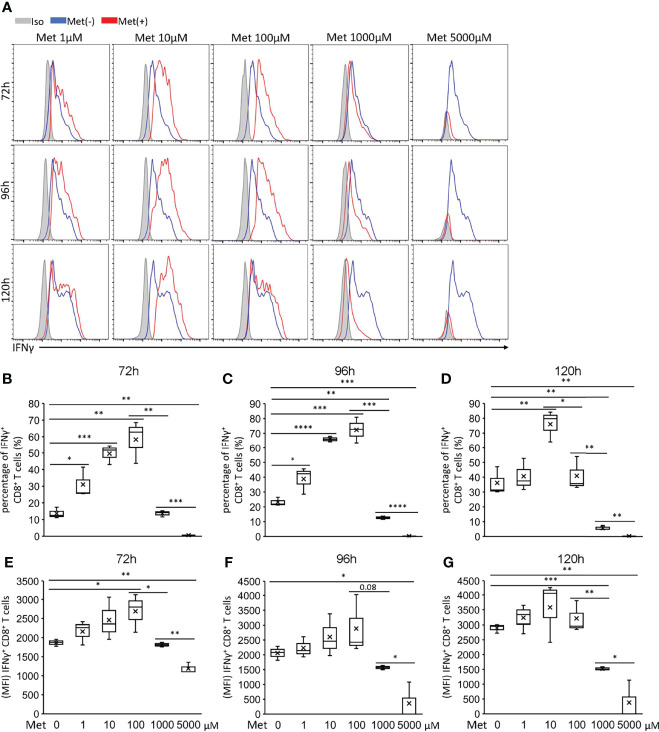
Met below 100 μM enhances IFNγ production by CD8^+^ T cells upon TCR stimulation. Splenic CD8^+^ T cells were cultured with the indicated concentrations of Met for 72, 96, and 120 h. The resulting cells were stimulated with PMA and ionomycin for 6 h, followed by the staining of intracellular IFNγ. **(A)** A representative flow cytometry plot showing the IFNγ levels in the CD8^+^ T cells. **(B–D)** The graph represents the percentage of the IFNγ^+^ CD8^+^ T cells at **(B)** 72 h, **(C)** 96 h, and **(D)** 120 h. **(E–G)** The graph represents the MFI of the IFNγ^+^ CD8^+^ T cells at **(E)** 72 h, **(F)** 96 h, and **(G)** 120 h. The graphs represent Mean ± SEM of the results of three independent experiments. Statistical analysis was performed by unpaired, two-tailed Student’s t-test (*P ≤ 0.05; **P ≤ 0.01; ***P ≤ 0.001; ****P ≤ 0.0001).

Next, we examined whether Met affected the IFNγ production by CD8^+^ T cells upon TCR stimulation. CD8^+^T cells isolated from mice spleen cells using magnet beads were stimulated with anti-CD3 Ab and anti-CD28 Ab for 72 to 120h in the presence of varying doses of Met at 0–5 mM to examine the IFNγ production. At 10 μM, Met markedly enhanced IFNγ production during the 72, 96, and 120h incubations (**Figure 2**). Although Met at 100 μM also enhanced IFNγ production during the 72 and 96h incubations, the enhancement declined at 120h (**Figure 2**). Importantly, Met at greater than 1 mM completely abolished the IFNγ production by CD8^+^ T cells such that IFNγ levels were even lower than those in the cells without Met (**Figure 2**). Similar results were observed on the expression of CD25, an activation marker of T cells, on the cell surface (**Figure S1A**) and on the production of TNFα (**Figure S1B**) of CD8^+^ T cells. The results indicated that Met at 10 μM, a physiologically relevant concentration, could enhance the IFNγ production by CD8^+^ T cells upon TCR stimulation and that Met at concentrations high enough to downregulate OCR of tumor cells, hampered the IFNγ production of CD8^+^ T cells.

### Enhanced IFNγ Production by Met-Treated CD8^+^ T Cells Is Abolished by a Mitochondrial ROS Scavenger

**Figure 3:**
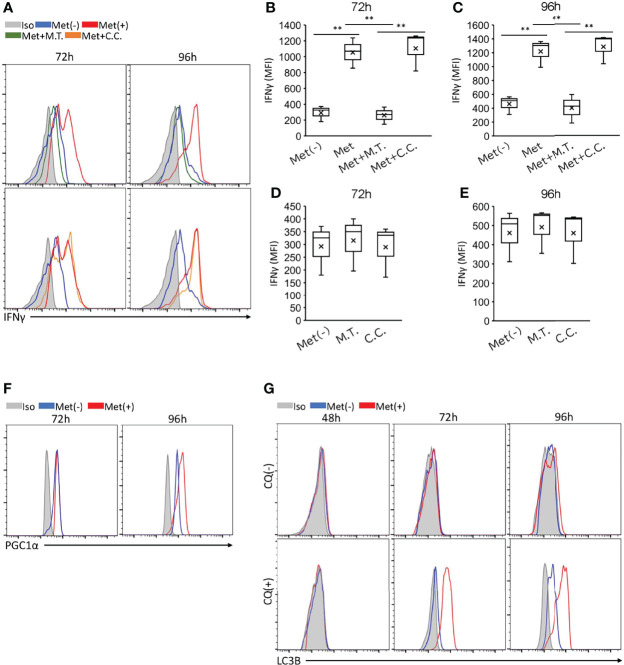
Met-induced enhancement of IFNγ, PGC1α, and LC3B of CD8^+^ T cells and the effects of inhibitors. Splenic CD8^+^ T cells were cultured with anti-CD3 and anti-CD28 Abs in the presence or absence of 10 μM Met for 48, 72 or 96 h. **(A–E)** The resulting cells were stimulated with PMA and ionomycin in the presence or absence of Met and inhibitors as indicated for 6h. **(A)** A representative flow cytometry plot showing IFNγ levels in the CD8^+^ T cells treated with or without Mito Tempo (M.T.) (upper panels), and Compound C (C.C.) (lower panels). **(B–E)** The graph represents the MFI of IFNγ levels at **(B, D)** 72 h and **(C, E)** 96 h. **(F)** A representative flow cytometry plot showing PGC1α levels in the CD8^+^ T cells that were cultured for 72 or 96 h with or without Met. **(G)** A representative flow cytometry plot showing LC3B levels in the CD8^+^ T cells that were cultured for 48,72, and 96h with or without treatment by Chloroquine (CQ) for last 6h. The graphs represent Mean ± SEM of the results of three independent experiments. Statistical analysis was performed by unpaired, two-tailed Student’s t-test (**P ≤ 0.01).

Recently, we observed that the enhanced IFNγ production by the CD8TILs in mice treated with Met was completely negated by the co-administration of MitoTEMPO, a mitochondrial ROS (mtROS) scavenger (11). Therefore, we examined the effect of MitoTEMPO on the *in vitro* IFNγ production by CD8^+^ T cells upon TCR stimulation with 10 μM Met. As expected, the enhanced IFNγ production was reduced by MitoTEMPO to the level of the CD8^+^ T cells without Met treatment (**Figure 3A**; upper panel, **Figures 3B, C**). However, compound C, an inhibitor for AMPK, did not suppress the IFNγ production of CD8^+^ T cells (**Figure 3A**; lower panel, **Figures 3B, C**), suggesting that Met stimulated IFNγ production of CD8^+^ T cells in a mtROS but not AMPK-dependent manner. It is of note that both MitoTEMPO and compound C had no inhibitory effect on IFNγ production of CD8^+^ T cells upon TCR stimulation without Met (**Figures 3D, E**). Concerning mitochondrial biogenesis, we examined the level of PGC1α in the cells. Met at 10 μM elevated the PGC1α levels in CD8^+^ T cells in the 96 but not 72h incubation (**Figure 3F**). As we previously found that autophagy was involved in the proliferation of CD8TILs in mice treated with Met (11), we checked the level of LC3B, an autophagy marker, in the CD8^+^ T cells with or without chloroquine (CQ), a blocker of the degradation of LC3B within autophagosomes. The accumulation of LC3B was evident at 72 h post culture, and its level was more pronounced in the 96-h incubation in the presence of CQ (**Figure 3G**). These findings, together with our previous findings (11), confirmed that Met stimulated the production of mtROS, IFNγ production, PGC1α expression, and autophagy.

### Differential Metabolic Pathways Required for Met-Induced IFNγ Production by CD8^+^ T Cells and CD8TIL

Next, we searched for the metabolic pathway utilized for the IFNγ production by the CD8^+^ T cells *in vitro* and the CD8TILs *ex vivo*. The translation of IFNγ in CD8^+^ T cells depends on the GAPDH in glycolysis (20), while the transcription of the IFNγ gene depends on phosphoenolpyruvate (PEP) (21), a metabolite in glycolysis that is essential in Ca^2+^ mobilization to promote NFAT translocation into the nucleus before IFNγ synthesis. The contribution of GAPDH to IFNγ production exclusively depends on glucose. However, PEP can be generated from either glucose or oxaloacetate in a PCK1-dependent manner (21). Oxaloacetate, a metabolite in the TCA cycle, may be generated from glucose, fatty acid, and glutamine/glutamate, suggesting that PEP generation, and hence IFNγ production, can depend on glycolysis, fatty acid oxidation (FAO), and glutaminolysis. These three mechanisms can fuel the TCA cycle in a process called anaplerosis.

**Figure 4:**
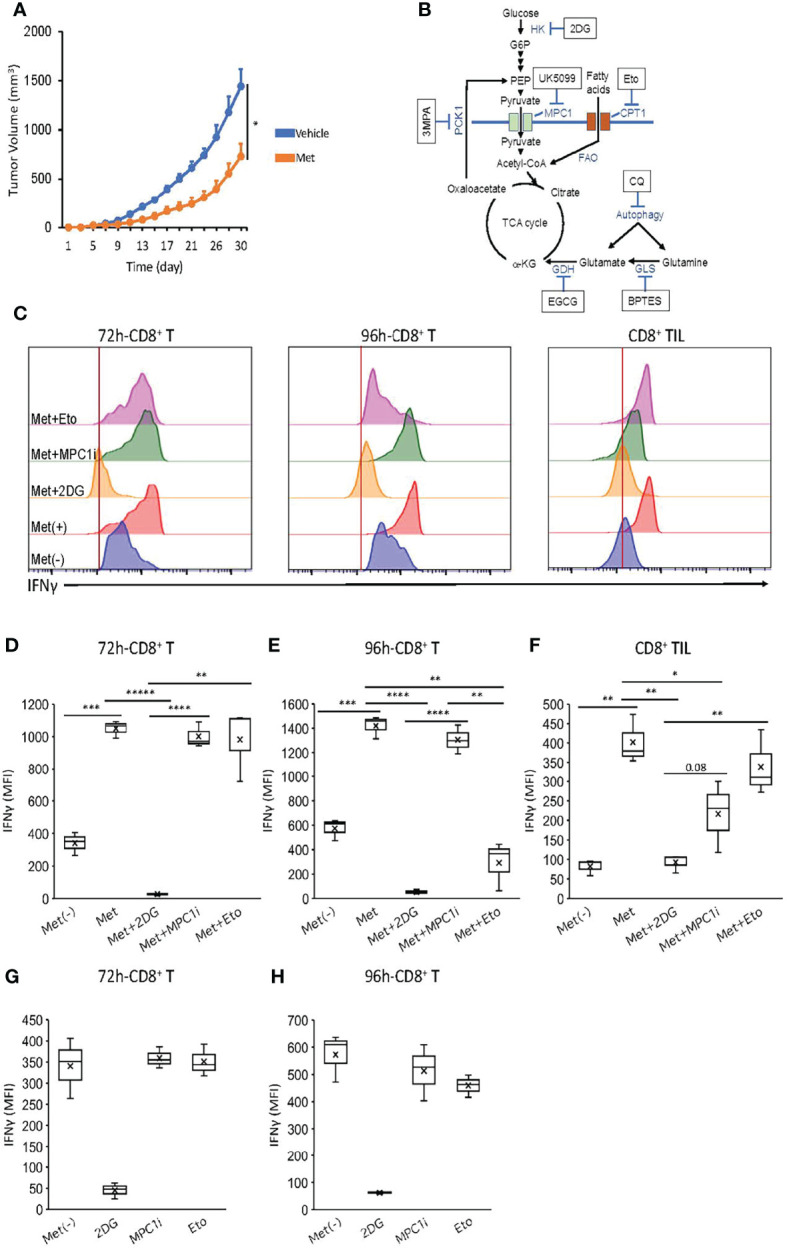
FAO is essential for IFNγ production by CD8^+^ T cells cultured for 96 but not 72h. For *in vitro* activated CD8^+^ T cell preparation, splenic CD8^+^ T cells were cultured as in Figure 3. For CD8TILs preparation, mice were treated with Met from day 7 post tumor inoculation, and on day 10, tumors were collected for flow cytometry analysis. The *in vitro* activated CD8^+^ T cells and CD8TIL were stimulated with PMA and ionomycin in the presence or absence of MPC1i (UK5099, a MPC1inhibitor) for 6h, and Etomoxir (Eto) and 2DG for 3h. **(A)** MO5 tumor growth curve of mice treated with or without Met. **(B)** The inhibition sites by the specific inhibitors. **(C)** The levels of IFNγ of *in vitro* activated CD8^+^T cells (72h, 96h) and CD8TILs treated with or without inhibitors as indicated. **(D–H)** The graph represents the MFI of IFNγ levels *in vitro* activated CD8^+^T at **(D, G)** 72h, **(E, H)** 96 h, and CD8TILs **(F)**. The graphs represent the mean ± SEM of the results of three independent experiments. Statistical analysis was performed by unpaired, two-tailed Student’s t-test (*P ≤ 0.05; **P ≤ 0.01; ***P ≤ 0.001; ****P ≤ 0.0001; *****P ≤ 0.00001).

To identify the metabolic (anaplerotic) pathways involved in IFNγ production, we generated effector CD8^+^ T cells *in vitro* upon TCR stimulation using Met. We also generated CD8TILs *ex vivo* on day 10 from the mice treated with Met from day 7 after inoculation with MO5 tumor cells. The *in vivo* experiment revealed a significant reduction of MO5 cell growth (**Figure 4A**). In addition, the metabolic pathways and the inhibition sites likely inhibited by the specific inhibitors were shown (**Figure 4B**). Thus, we evaluated the IFNγ production by the CD8^+^ T cells *in vitro* and by the CD8TILs *ex vivo* in the presence of the metabolic inhibitors.

We found that only 2DG (a hexokinase inhibitor) blocked the IFNγ production by CD8^+^ T cells upon TCR stimulation with Met on day 3 (72h), and etomoxir (a CPT1 inhibitor blocking FAO) significantly blocked the IFNγ production by CD8^+^ T cells on day 4 (96h) (**Figures 4C–E**), suggesting that on day 3, the CD8^+^ T cells depended on glycolysis only, while on day 4 they depended on both glycolysis and FAO for IFNγ production. Furthermore, in the case of the CD8TILs, 2DG and MPC1 (blocking pyruvate entry to mitochondria), but not etomoxir, significantly blocked IFNγ production, suggesting that pyruvate generation from glucose and its translocation to the mitochondria are involved in the IFNγ production by CD8TILs. It is of note that IFNγ production by CD8^+^ T cells upon TCR stimulation without Met was not sensitive to inhibition of etomoxir (**Figures 4G, H**), which was in contrast to the results of CD8^+^ T cells with Met (**Figure 4E**).

**Figure 5:**
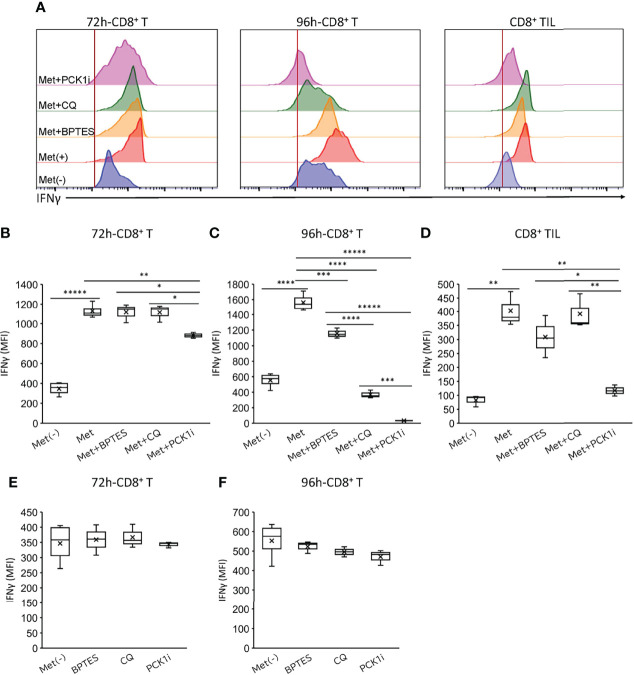
Differential metabolisms required for IFNγ production by *in vitro* activated CD8^+^T cells and CD8TILs. The *in vitro* activated CD8^+^ T cells and CD8TILs were prepared as in Figure 4. The cells were stimulated with PMA and ionomycin in the presence or absence of 3MPA (PCK1i), chloroquine (CQ), and BPTES for 6h. **(A)** The levels of IFNγ of *in vitro* activated CD8^+^T cells (72h, 96h) and CD8TIL treated with or without inhibitors as indicated. **(B–F)** The graph represents the MFI of IFNγ levels *in vitro* activated CD8^+^ T cells at **(B, E)** 72h, **(C, F)** 96 h, and CD8TILs **(D)**. The graphs represent the mean ± SEM of the results of three independent experiments. Statistical analysis was performed by unpaired, two-tailed Student’s t-test (*P ≤ 0.05; **P ≤ 0.01; ***P ≤ 0.001; ****P ≤ 0.0001; *****P ≤ 0.00001).

We further investigated if autophagy, glutaminolysis, and PCK1-dependent pathways were involved in IFNγ production. On day 3, we found in CD8^+^ T cells a weak inhibition by 3MPA (PCK1 inhibitor) and no inhibition by BPTES (an inhibitor of GLS blocking the glutamine-to-glutamate conversion) and CQ, suggesting that glutaminolysis and autophagy were not involved in IFNγ production by day 3 in CD8^+^ T cells (**Figures 5A, B**). However, profound inhibition by 3MPA and CQ and a weak but significant inhibition by BPTES were observed on day 4 in the CD8^+^ T cells (**Figures 5A, C**), suggesting that on day 4, CD8^+^ T cells depended on the PCK1-dependent PEP production from oxaloacetate and autophagy involving glutaminolysis for IFNγ production. It is of note that IFNγ production by CD8^+^ T cells upon TCR stimulation without Met was not blocked by 3MPA and CQ (**Figures 5E, F**), in contrast to the results of CD8^+^ T cells with Met (**Figure 5C**). Intriguingly, 3MPA significantly blocked the IFNγ production by CD8TILs (**Figures 5A, D**). As CQ and BPTES did not block the IFNγ production by CD8TILs, autophagy and glutaminolysis were not involved, unlike the results on the CD8^+^ T cells on day 4. Together with the results in **Figure 4F**, CD8TILs appear to depend on both glucose-dependent anaplerosis and PCK1-dependent PEP production from oxaloacetate for IFNγ production.

### Glucose Prevents the Metabolic Switch From Glycolysis to FAO for IFNγ Production by CD8^+^ T Cells

**Figure 6:**
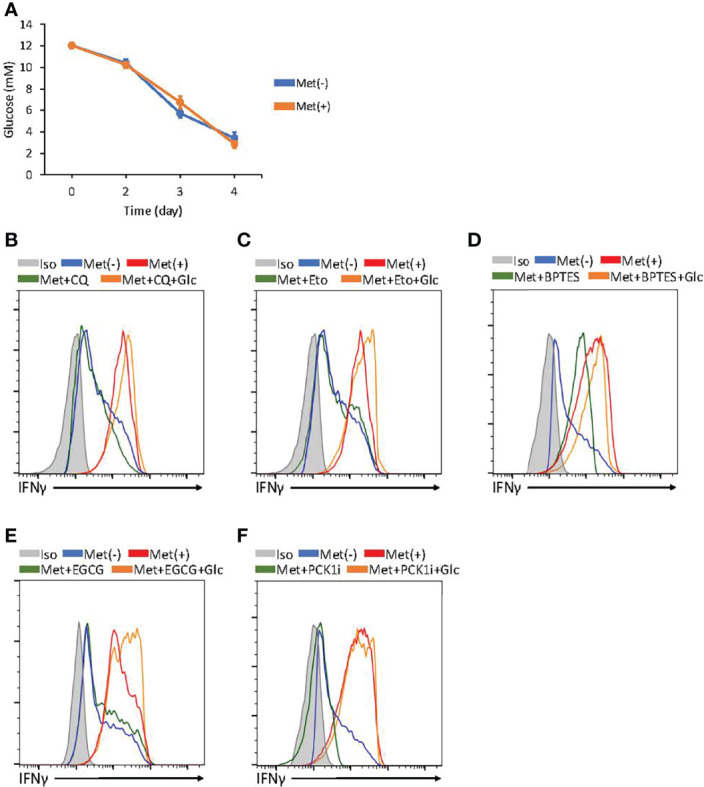
Glucose concentration determines metabolism required for IFNγ production by *in vitro* activated CD8^+^ T cells. **(A)** The graphs represent the glucose concentrations in the supernatant of CD8^+^ T cell cultured with or without Met. (B–F) *In vitro* activated CD8^+^ T cells at 72h were further incubated with or without supplementation of glucose for additional 24h and the resulting cells were treated with PMA and ionomycin in the presence or absence of inhibitors, as indicated in CQ **(B)**, Etomoxir **(C)**, BPTES **(D)**, epigallocatechin gallate (EGCG) **(E)**, and 3MPA(PCL1i) **(F)**. A representative flow cytometry plot showing the *in vitro* IFNγ levels in the CD8^+^ T cells at 96h. The graphs represent the mean ± SEM.

On day 3, the CD8^+^ T cells exclusively depended on glycolysis for IFNγ production; in contrast, on day 4, the CD8^+^ T cells depended on FAO, autophagy, and glutaminolysis in a PCK1-dependent manner. We wondered the differential metabolic requirement between days 3 and 4 CD8^+^ T cells was due to the difference in glucose concentrations in the culture supernatant. Therefore, we monitored the glucose concentration in the culture supernatant. We detected the glucose concentration at 12 mM on day 0, which declined to 6 mM on day 3 and 3–4 mM on day 4 (**Figure 6A**). There was no significant difference in the glucose concentration in the supernatant of the cells with or without Met (**Figure 6A**). Therefore, the glucose concentration at 3–4 mM might be a point where the metabolism of CD8^+^ T cells switched to FAO for IFNγ production in the presence of Met. If so, glucose supplementation to the culture would keep the IFNγ production exclusively dependent on glycolysis. Thus, we added glucose back to the culture to increase the glucose concentration to 12 mM for the final 24h on day 4, then IFNγ production was evaluated in the presence of metabolic inhibitors. We found that IFNγ production was decreased by CQ, etomoxir, BPTES, EGCG (an inhibitor of GDH blocking the glutamate-to-αKG conversion), and PCK1 inhibitor; on the other hand, IFNγ production was completely restored by glucose supplementation (**Figures 6B–F**). These data suggest that glucose is a critical factor for determining the metabolic pathways required for IFNγ production by CD8^+^ T cells *in vitro*.

## Discussion

The current study reports for the first time the direct effect of Met on the IFNγ production by CD8^+^ T cells. OCT1, a transporter for Met, was shown to be significantly enhanced in CD8^+^ T cells by TCR stimulation *in vitro* (11), suggesting that the direct effect of Met was only on activated T cells and not on naive T cells. This finding is consistent with the *in vivo* observation that Met administration activates IFNγ production of CD8TILs but not CD8^+^T cells in the lymph node and the spleen (11). The suitable concentration of Met for IFNγ production by CD8^+^ T cells is less than 100 μM and ideally 10 μM, a physiologically relevant concentration. Our findings may contribute to the understanding of Met’s antineoplastic effect on patients with diabetes whose plasma concentration of Met is around 10 μM; thus, the effect may be mediated by the immunosurveillance mechanism. Our findings are consistent with another study, which found that the direct effect of 10 μM Met on NK cells to produce IFNγ might be involved in immunosurveillance (22). Met at 1 mM or higher inhibits OxPhos while stimulating glycolysis of tumor cells. It may imply that Met at 1mM enhances the Warburg effect, which may have a risk to give preferential effect for the tumor to survive under certain conditions, even if such a high concentration of Met was possible *in vivo*.

T cells almost do not produce IFNγ when the glucose concentration is less than 1 mM due to the GAPDH-mediated- inhibition of IFNγ synthesis (20) and the reduced production of PEP, which is essential for the transcription of the IFNγ gene (21). The glucose concentration in our *in vitro* experiments was found to be 3–12 mM, more than sufficient for IFNγ production. However, the metabolic pathways involved are different for 3mM and 12 mM glucose. Thus, CD8^+^ T cells shift their dependence on glycolysis to FAO or autophagy-dependent glutaminolysis or both, followed by PCK1-dependent IFNγ production, as the glucose concentration declined to 3 mM on day 4 from 6 mM on day 3. The flexible metabolism of CD8^+^ T cells is indeed the case in the presence of Met and is not observed without Met. Met-dependent PGC1α synthesis and autophagy induction became apparent after 96 h of incubation, coinciding with the metabolic switch to FAO from glycolysis. Thus, mitochondrial activation by anaplerosis with fatty acids or glutamine/glutamate or both might be a key step for the metabolic reprogramming in CD8^+^ T cells.

**Figure 7:**
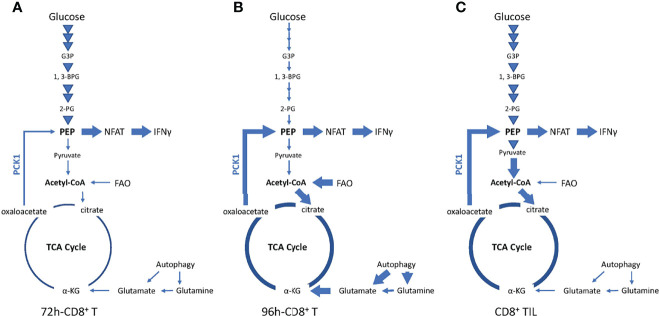
Metabolic preference of *in vitro* activated CD8^+^ T cells and CD8TILs for Met-induced enhancement of IFNγ production. **(A)** *In vitro* activated CD8^+^ T cells at 72 h exclusively depends on glycolysis. **(B)** *In vitro* activated CD8^+^ T cells at 96 h depends on FAO, glutaminolysis/autophagy, and PCK1. **(C)** CD8TILs depends on anaplerosis from glucose metabolism and PCK1.

Quite surprisingly, the *ex vivo* IFNγ production by CD8TILs appeared to depend on glycolysis-derived pyruvate that would be converted to oxaloacetate in the TCA cycle, followed by PCK1-dependent IFNγ production (**Figure 7**). This pathway might activate both glycolysis and OxPhos in CD8TILs, enhancing IFNγ production and causing cell proliferation, respectively. In fact, our previous study identified a novel role of Met in stimulating the production of mtROS. mtROS stimulates glycolysis to produce IFNγ while promoting cell proliferation *via* the activation of the Nrf2/mTORC1/p62 axis in CD8TILs (11). CD8TILs metabolism may be unique to Met’s treatment because Met upregulates Glut-1 level on the surface of the CD8TILs; moreover, it elevates the glucose concentration in a tumor, likely through the downregulation of the glycolysis of tumor cells in an IFNγ-dependent manner (11). However, CD8TILs metabolism was only examined in MO5 melanoma cells in this study; it is necessary to investigate this mechanism in other tumor models.

The conflicting argument that glycolysis and OxPhos are both most important for effector T cells to fight cancer (23–26), may be two sides of the same coin. The effector function, such as IFNγ production of CD8TILs, is heavily dependent on glycolysis. The analysis of CD8TILs for their IFNγ production revealed the importance of glycolysis over OxPhos. On the other hand, OxPhos is more important in effector T cells that will be adoptively transferred to tumor-bearing mice (26, 27) because the transferred T cells must adapt to a new environment, thus requiring elevated OxPhos in the healthy mitochondria. After ensuring survival in tumor tissues, the transferred T cells can proliferate while fighting cancer. In this phase, Oxphos for cell survival and proliferation and glycolysis for T cell function become important.

Similarly, Met’s effect on effector T cells may have different aspects. We previously observed that treatment with Met for a short period of approximately 6 h conferred a better migration or expansion ability, or both, on tumor-specific, naive CD8^+^ T cells in the tumor after their adoptive transfer to tumor-bearing mice, compared with those of the CD8^+^ T cells without Met treatment. This effect was blocked by the treatment of T cells with compound C before their adoptive transfer (10); thus, this effect might be AMPK-dependent. However, the specificity of compound C is broad beyond AMPK. Met appeared to protect the transferred T cells from apoptosis after their migration into the tumor in this case. The current study, however, has revealed the importance of glycolysis and pyruvate-dependent anaplerosis for IFNγ production by CD8TILs *ex vivo*; the effect is sensitive to MitoTEMPO but not compound C. Concerning the involvement of AMPK for T cell effector function, a previous report suggest that LKB1-AMPK axis negatively regulates T cell function (28), which might be consistent with our results in the current study. Therefore, the AMPK requirements of effector T cells for antitumor response may vary depending on the experimental conditions.

## Materials and Methods

### Metformin, Inhibitors

Metformin hydrochloride (Tokyo Chemical Industry), 2-deoxy-D-glucose (Sigma-Aldrich), Etomoxir (Selleck Chemicals), ethyl sulfide (BPTES, Sigma-Aldrich), Chloroquine hydrochloride (Sigma-Aldrich), UK5099 (Sigma-Aldrich), epigallocatechin gallate (Tokyo Chemical Industry), 3MPA (Sigma-Aldrich), MitoTempo (Sigma-Aldrich), Compound C (Sigma-Aldrich) were purchased.

### Mice

Female C57BL/6 (B6) mice (SLC, Shizuoka, Japan) were used for all experiments. All mice were maintained under specific pathogen-free conditions in the animal facility of Okayama University. This study was approved by The Institutional Animal Care and Use Committee of Okayama University Graduate School of Medicine (OKU-2018224).

### Tumor Cell Lines, Cell Culture

B6 OVA gene-transduced B16 melanoma MO5 cells were used for tumor assays (10). Meanwhile, 3LL cells (kindly provided by Dr. H. Yagita at the Juntendo University of Medicine in Tokyo, Japan) were cultured in a 96-well plate with 100 μl of RPMI supplemented with 10% fetal calf serum (FCS), L-glutamine, 2-ME, sodium pyruvate, and NEAA, treated with metformin at 0, 1, 10, 100, 1000, or 5000 μM for 48h, and used for extracellular flux analysis.

### Extracellular Flux Analysis

The oxygen consumption rate (OCR) and extracellular acidification rate (ECAR) of MO5 and 3LL cells were measured in XF media (nonbuffered RPMI 1640 containing 10 mM glucose, 2 mM L-glutamine, and 1 mM sodium pyruvate) under basal conditions and in response to mitochondrial inhibitors, 4 μM oligomycin (Sigma-Aldrich), 8 μM FCCP (Sigma-Aldrich), or 100 mM 2DG (Sigma-Aldrich) only or 1 μM rotenone (Sigma-Aldrich) combined with 1 μM antimycin A (Sigma-Aldrich) and 100 mM 2DG (Sigma-Aldrich) using an XFe96 Extracellular Flux Analyzer (Agilent Technologies). Cell count evaluated for the metabolism were normalized using a Cytation1 Cell Imaging Multi-Mode Reader (BioTek).

### CD8^+^ T Cell Purification and Expansion

CD8^+^ T cells were purified from the spleen using magnetic separation (Miltenyi Biotec), cultured with 1 μg/ml plate-bound anti-CD3 (eBioscience) and 2 μg/ml soluble anti-CD28 (eBioscience), in a 96-well plate with 200 μl of RPMI supplemented with 10% FCS, 2 mM L-glutamine, 5 x 10^-5^M 2-mercaptoethanol, 1 mM sodium pyruvate, and 0.1 mM non-essential amino acids. 5 x 10^4^ CD8^+^ T cells were cultured in each well. Cell blast formation occurred 24 h after cell culture. Cell numbers became 6.5 x 10^5^ after 72-hour-culture and dead cells were observed below 10% under microscope by Trypan blue staining. For the metformin-treated group, cells were treated with metformin at 0, 1, 10, 100, 1000, or 5000 μM. Cells were collected at 48, 96, 120h for extracellular flux analysis or flow cytometry.

### Tumor Engraftment and *In Vivo* Metformin Treatment

For melanoma cell engraftment, 2 × 10^5^ MO5 cells were suspended in 200 μl of RPMI and injected subcutaneously into the right side of the back of a wild-type C57BL/6 mouse. Then, 7 days after engraftment, 5 mg/mL metformin was administered perorally. Next, 3 days after metformin treatment, tumors were collected for FACS analysis. For the tumor volume experiment, the metformin treatment lasted 30 days. The long (a) and short (b) tumor axes were measured using a pair of Vernier calipers to calculate the mean diameter, whereas tumor volume (V) was calculated as V = ab^2^/2.

### Tumor-Infiltrating Lymphocytes Collection

Tumors were dissected from mice and minced in phosphate-buffered saline (PBS) supplemented with 5 mM EDTA and 2% FCS. Cells were harvested from the minced tumor tissues using the BD Medimachine system. All cells, including TILs and tumor cells, were stained with fluorescently-labeled antibodies (see below) and subjected to flow cytometry.

### Flow Cytometry and Intracellular Cytokine Staining

For intracellular IFNγ or TNFα measurement, cells were incubated with or without metformin and/or an inhibitor, thus, 2DG (500 mM), Etomoxir (10 μM), BPTES (20 μM), Chloroquine (50 μM), UK5099 (10 μM), EGCG (50 μM), 3MPA (500 μM), MitoTempo (50 μM), Compound C (10 μM), in the presence of 1.25 ng/mL Phorbol 12-myristate 13-acetate (PMA, Sigma-Aldrich), 50 nM ionomycin (Sigma-Aldrich) and GolgiStop the Protein Transport Inhibitor (containing Monensin, BD Bioscience) for 6h (Etomoxir only for last 3h). After incubation, cells were collected for surface staining with CD3 (17A2) and CD8 (53-6.7) Abs in PBS supplemented with 5 mM EDTA and 2% FCS, in the dark for 30 min at 4°C, followed by fixation and permeabilization using Fixation/Permeabilization kit (BD Biosciences) in the dark for 30 min at 4°C. Then intracellular IFNγ (XMG 1.2) or TNFα (MP6-XT22) Ab probed for cells in the dark for 30 min at 4° C. For LC3B measurement, cells were collected for CD8 and CD3 surface staining, LC3B (Novus Biologicals) antibodies staining was performed using a Fixation/Permeabilization kit (BD Biosciences). For PGC1α measurement, cells were collected for CD8 and CD3 surface staining. Staining for transcription factors was performed with a Transcription Factor Buffer Set (BD Pharmingen™) with PGC1α (Novus Biologicals) antibody. For CD25 measurement, cells were collected and performed CD8, CD3 and CD25 (PC61) surface staining in the dark for 30 min at 4° C.

### Measurement of Glucose in CD8^+^ T Cells Supernatants

Cell culture Supernatants were collected 72, 96, and 120h after culturing CD8^+^ T cells and diluted 1:20 for analysis. A Glucose Assay Kit-WST (DojinDo Laboratories) was used to measure the glucose concentrations.

### Glucose Supplement for T Cells

After 72 h CD8^+^ T cells culture, D-(+)-glucose (Fujifilm Wako Pure Chemical Corporation) in 10 μl of PBS was added to medium to achieve 12 mM glucose concentration in culture medium, consisting with the concentration at 0 h. Meanwhile, 10 μl of PBS was added to the negative control group. Intracellular cytokine staining and flow Cytometry were performed 24h after the glucose supplement.

## Data Availability Statement

The original contributions presented in the study are included in the article/**Supplementary Material**. Further inquiries can be directed to the corresponding author.

## Ethics Statement

This study was approved by The Institutional Animal Care and Use Committee of Okayama University Graduate School of Medicine.

## Author Contributions

RC performed the experiments and wrote some parts of paper. MN designed the all experiments and supervised the experimental procedures. NY maintained the mice used in the experiments. MN, MT, WZ, and IK contributed to the extensive discussion throughout the entire experiments and proof reading of the manuscript. HU supervised the project, designed the experiments, and wrote the paper. All authors contributed to the article and approved the submitted version.

## Funding

This study was supported by grants to HU from JSPS KAKENHI Grant number 18H04033 and 17K19598.

## Conflict of Interest

The authors declare that the research was conducted in the absence of any commercial or financial relationships that could be construed as a potential conflict of interest.

## Publisher’s Note

All claims expressed in this article are solely those of the authors and do not necessarily represent those of their affiliated organizations, or those of the publisher, the editors and the reviewers. Any product that may be evaluated in this article, or claim that may be made by its manufacturer, is not guaranteed or endorsed by the publisher.
