# Supplementary figures and images for: Nutrient Condition in the Microenvironment Determines Essential Metabolisms of CD8+ T Cells for Enhanced IFNγ Production by Metformin
Source: Front Immunol. 2022 Jun 29;13:864225. doi: 10.3389/fimmu.2022.864225 (PMC9277540; doi:10.3389/fimmu.2022.864225)

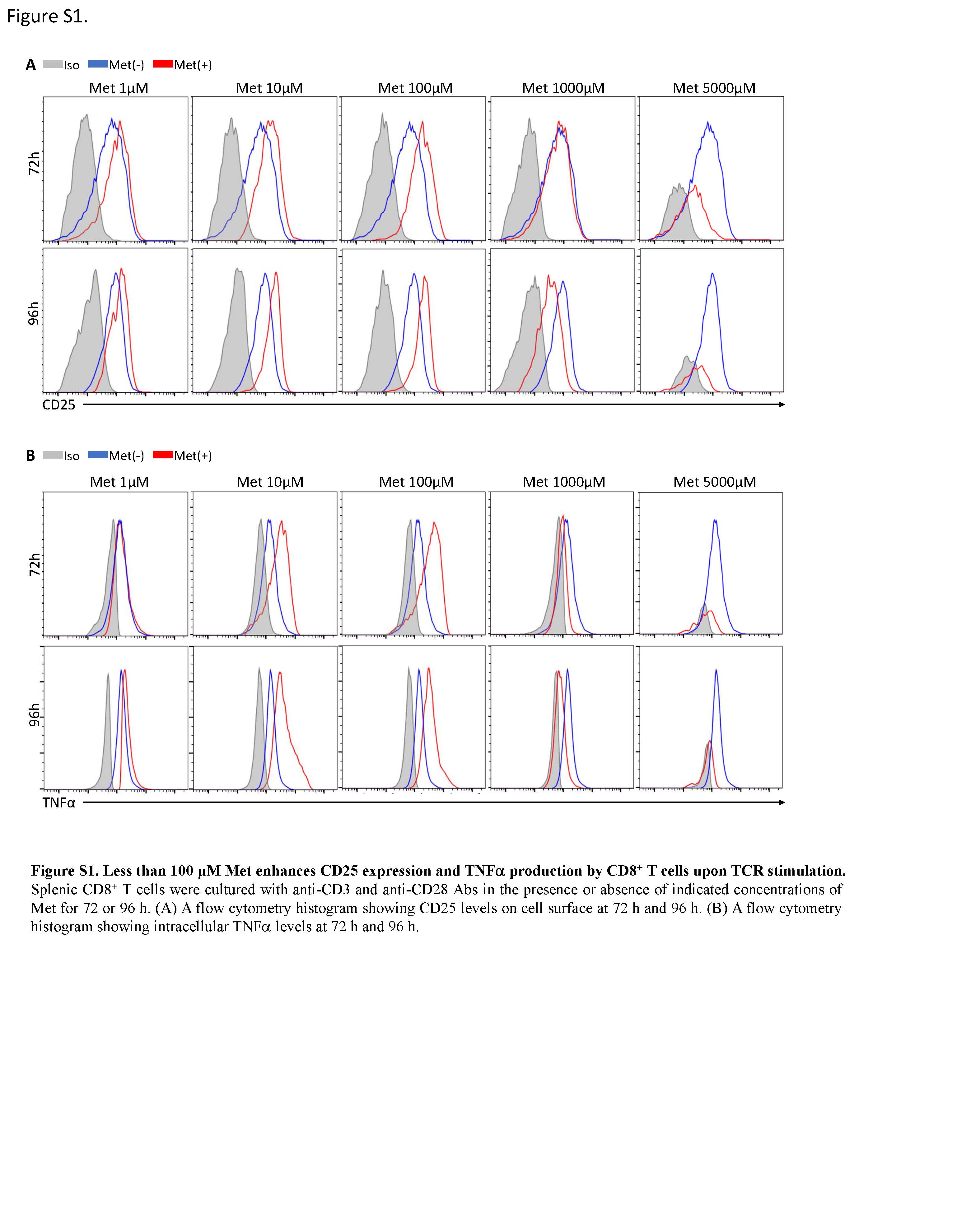

Supplement: Supplementary file 1 [file Image_1.jpeg]
